# Suture Slippage Is Influenced by Suture Type and Bone Density in a Knotless Lateral Row Rotator Cuff Repair: A Biomechanical Analysis

**DOI:** 10.1002/ars2.70083

**Published:** 2026-09-25

**Authors:** Kushalappa Subbiah, Matthew Donaldson, Cameron Stephen, Allan Young, Benjamin Cass, Elizabeth C. Clarke, Margaret M. Smith

**Affiliations:** ^1^ Sydney Shoulder Research Institute Sydney New South Wales Australia; ^2^ Faculty of Medicine and Health School of Medical Sciences Sydney Musculoskeletal Health, University of Sydney Sydney Australia; ^3^ Kolling Institute Northern Sydney Local Health District Murray Maxwell Biomechanics Laboratory Sydney Australia

## Abstract

**Purpose:**

To evaluate the influence of suture material and bone density on suture slippage in knotless lateral row anchors used in rotator cuff repair.

**Methods:**

Analysis of 3 different suture materials loaded into a single lateral row anchor and tested in 2 different densities (osteoporotic and normal) of Sawbone models was conducted. Testing was carried out using perpendicular traction of loaded sutures in a load cell with assessment of maximum force (N) and displacement (mm). After testing completion, the blocks were visually analyzed, and cut‐through length (mm) was measured.

**Results:**

Suture type (*P *< .001) and bone density (*P *< .001) have a significant effect on maximum force tolerance by the construct. FiberTape can withstand a much higher maximum force irrespective of bone densities. Displacement data collected from the load cell shows that suture type (*P *< .001), but not bone density (*P* = .228), plays a significant role, with FiberTape having a comparatively lower displacement. Evaluation of cut‐through data shows that bone density (*P* = .006), but not suture type (*P* = .94), has a significant effect on cut‐through.

**Conclusions:**

Suture slippage in knotless lateral row fixation is significantly influenced by the suture material and the underlying bone density. Constructs using high‐strength suture tape showed higher load‐to‐failure and lower displacement compared with conventional sutures.

**Clinical Relevance:**

Our study shows that the weakest link in a knotless repair is the lateral row, and this can be influenced by the type of suture used in the construct along with the density of bone.

## Graphical abstract

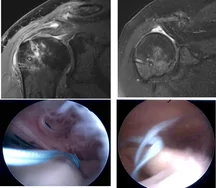

Arotator cuff tear is a commonly occurring pathology of the shoulder joint which is intrinsically associated with pain, weakness, and disability of the shoulder. Rotator cuff repair has been shown to be an effective treatment option in restoring the muscle‐tendon‐bone unit and in turn the function of the shoulder joint.^1^ Historically, the results of rotator cuff repair have varied widely with failure rates reported from 11% to 94% in the literature.^2^ Failure rates have shown no significant difference based on the method of repair, i.e., open/arthroscopic, single row/double row, and knot/knotless.^3^, ^4^, ^5^, ^6^, ^7^, ^8^, ^9^, ^10^, ^11^, ^12^ Several studies have looked to identify and in turn attempted to quantify the risk factors associated with repair failure.^13^, ^14^, ^15^ This has led to the establishment of a scoring system which takes into consideration several patient‐related factors to predict the failure of a rotator cuff repair.

The various predictive factors which are taken into consideration are age, size of the tear, level of fatty infiltration, degree of tendon retraction, bone density, and level of activity. These factors are assigned scores and used to calculate the Rotator Cuff Healing Index.^16^ Bone density has been cited as an independent factor influencing the healing of the rotator cuff repair, and the same was noted by Chung et al.^15^ According to Gerber et al.,^17^ the ideal repair should have high initial fixation, minimal gap formation, and mechanical stability that is sufficient until the tendon‐to‐bone healing is completed.

Cho et al.,^18^ in their study of repair integrity, noted 2 broad methods of failure based on location in relation to the medial row anchors. They noted a lateral failure (type 1) and a medial failure (type 2); systematic reviews have shown type 1 repair failures to occur more in association with a knotless double row repair and type 2 more in association with a knot‐tied medial row.^19^

Kim et al.,^20^ in their study of postoperative ultrasounds of a cuff repair, noted medial migration of sutures from the medial anchors because of suture pullout from the lateral row. They noted that most slippage occurred within the initial 2 months of the repair, which coincides with the critical period of tendon‐to‐bone healing and subsequent enthesis formation for a successful rotator cuff repair.^21^

This pullout/slipping of sutures from the lateral row and loss of tendon‐bone contact over the footprint is proposed to be the reasoning behind type 1 repair failures.^4^, ^22^, ^23^, ^24^ Intraoperative observations of suture‐anchor‐bone interaction, retrospective analysis of repair failures, and literature of suture slippage from the lateral row in a knotless repair led to our hypothesis; osteoporotic bone increases the extent of suture slippage, and a combination of osteoporotic bone with different sutures allows for different degrees of suture slippage. We believe that a combination of weak bone and “slippery” sutures can propagate to failure of the repair in the early postoperative period (Figure 1). The purpose of this study was to evaluate the influence of suture material and bone density on suture slippage in knotless lateral row anchors used in rotator cuff repair. Our hypothesis was that suture type and bone density influence the integrity of the lateral row anchor construct.

**FIGURE 1:**
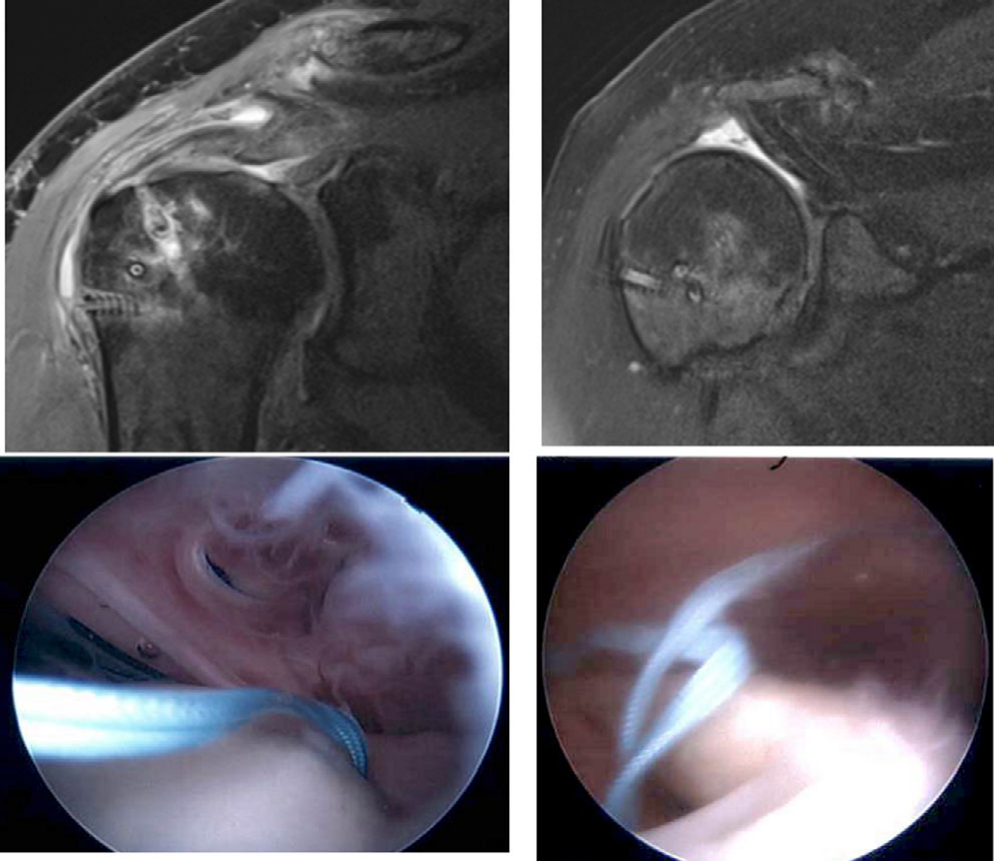
Magnetic resonance imaging: T2‐weighted sagittal images showing a rotator cuff repair failure with suture limbs in continuity with the tendon. Intraoperative images: posterior view of the subacromial space of 2 separate patients showing rotator cuff repair failure caused by suture slippage from a lateral row anchor.

## METHODS

### Experimental Procedure

Sawbone blocks (Sawbones, Vashon, WA) of 2 densities were chosen to represent normal bone (0.16 g/cc) and osteoporotic bone (0.08 g/cc).^25^ The bones were cut into equal blocks measuring 60 × 60 × 40 mm. Testing was done with 3 routinely used suture types—FiberWire, SutureTape (ST), and FiberTape (Arthrex, Naples, FL). The testing cycles included 6 cycles for each bone/suture combination; this was chosen to reduce the possible error from variation and provide a more accurate data set.

A fixed working length was chosen for the sutures and kept consistent along all testing cycles. The SwiveLock anchors (Biocomposite 5.5 × 19.1 mm; Arthrex) were loaded and then introduced into the pilot hole made in the block using the company‐provided punch. The suture limbs were then tensioned as normal during a rotator cuff repair, and the lateral row anchor was screwed in. Sutures were then cut flush with the bone. To recreate the arthroscopic environment, the entire setup was done submerged in a tub with saline. This setup was then mounted and tested in the load cell.

After completion of the testing protocol on the load cell, the blocks were retrieved for visual assessment, and cut‐through of the sutures was measured. Using a vernier calliper; measuring from the centre of the anchor out towards the most prominent point of cut‐through in the Sawbone block.

### Biomechanical Testing Setup

**FIGURE 2:**
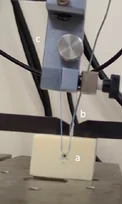
The testing setup: (a) Sawbone block with SwiveLock suture anchor. (b) Suture limbs leading away from the anchor perpendicular to the surface of the bone block, fixed loop length wrapped around load cell mount. (c) Load cell with custom mount. Mounting was done after insertion of the anchor in normal saline.

The loaded block was then transferred onto the hold of the load cell, and using a custom mount, the suture loop was held in the load cell (Instron DYNACELL 8338C) and preloaded to 5 N. At this point, video analysis was started to provide additional assessment of the testing unit. The load cell was then run through the testing cycle and data was collected (Figure 2). The variables we wanted to compare were the suture materials and bone density while maintaining consistency in the lateral row anchor design.

### Biomechanical Testing Protocol

The protocol comprised preloading and load‐to‐failure biomechanical testing. The vector of force was directed perpendicular to the anchor. The preloading stage began with gradual tensioning of the sutures at 0.5 mm/s intervals until a preload of 5 N was achieved. After preloading, the load was increased at 1 mm/s until load to failure occurred. Failure was defined as an abrupt drop in force curve/visible anchor pullout from the bone block.

## RESULTS

Data collected from the testing protocol included maximum force (N), displacement (mm), and cut‐through (mm). The collected data was then tested and found to approximate a normal distribution. Hence, liner regression and *t* test were used to test for differences between sutures and bone density for all dependent variables. (*The adjusted *R* ^2^ is the coefficient of determination and is defined as the proportion of the variation in the dependent variable that is explained by the independent variables in the model.)

### Maximum Force (N)

**FIGURE 3:**
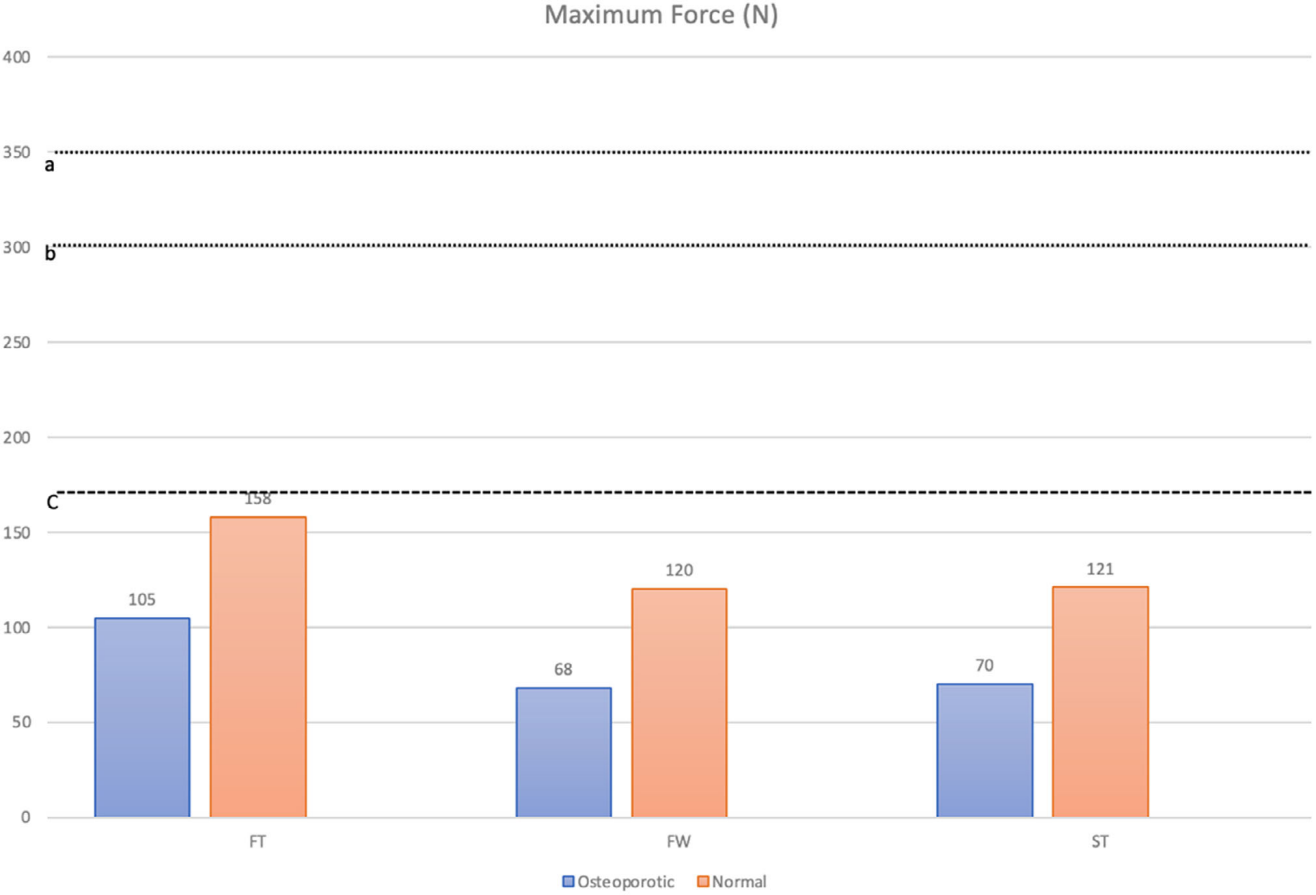
Bar graph showing the mean maximum force (N) tolerance of each suture in different block densities. Additional reference lines: (a) force at which the high‐strength braided polyblend UHMWPE sutures fail.^5^ (b) Force of supraspinatus contraction.^11^ (c) Resistance beyond which sutures cut out of rotator cuff tendon.^4^ (FT, FiberTape; FW, FiberWire; ST, SutureTape.)

Analyzing maximum force until failure, both suture type (*P* < .001) and bone density (*P* < .001) have a significant effect on maximum force tolerance by the construct (regression *P* < .001, *adjusted *R* ^2^ = 0.668). As shown in Table 1, all suture types were able to withstand a much lower maximum force when used in an anchor placed in bone with low density. Additionally, it can be seen when comparing suture types (Table 2 and Figure 3) that FiberTape is able to withstand a much higher maximum force irrespective of bone densities.

**TABLE 1:** Maximum Force (N): Results (Mean ± SD) With Significance Between Bone Densities

Bone Density	Low Density	High Density	*P* Value
Suture Type	Mean ± SD (n = 6)	Mean ± SD (n = 6)	
FT	105 ± 16	158 ± 33	.005
FW	67.6 ± 8.0	120 ± 26	<.001
ST	69.8 ± 4.2	121 ± 16	<.001

FT, FiberTape; FW, FiberWire; SD, standard deviation; ST, SutureTape.

**TABLE 2:** Maximum Force (N): Differences Between Sutures at Each Bone Density

Bone Density	Low Density	High Density
Suture Comparison	*P* Value	*P* Value
FT vs FW	<.001	.045
FW vs ST	.55	.92
ST vs FT	<.001	.030

FT, FiberTape; FW, FiberWire; ST, SutureTape.

### Displacement (mm)

On analyzing the displacement data collected from the load cell, we see that suture type (*P* < .001), but not bone density (*P* = .228), had a significant effect on displacement (regression *P* < .001, *adjusted *R* ^2^ = 0.460). FiberTape shows a difference in displacement between bone densities, with a higher displacement in lower bone density (Table 3). When compared with FiberWire and ST, it has a lower displacement in both tested bone densities (Table 4).

**TABLE 3:** Displacement (mm): Results (Mean ± SD) and Differences Between Bone Densities

Bone Density	Low Density	High Density	*P* Value
Suture Type	Mean ± SD (n = 6)	Mean ± SD (n = 6)	
FT	21.1 ± 1.8	17.8 ± 2.3	.026
FW	27.2 ± 2.3	25.9 ± 3.5	.48
ST	26.4 ± 1.6	26.6 ± 2.9	.88

FT, FiberTape; FW, FiberWire; SD, standard deviation; ST, SutureTape.

**TABLE 4:** Displacement (mm): Differences Between Sutures by Bone Density

Bone Density	Low Density	High Density
Suture Comparison	*P* Value	*P* Value
FT vs FW	<.001	.002
FW vs ST	.51	.73
ST vs FT	<.001	<.001

FT, FiberTape; FW, FiberWire; ST, SutureTape.

## DISCUSSION

Our results show a positive correlation between suture slippage and the type of suture used along with density of bone. Suture slippage as reported by Hayeri et al.^22^ and studied by Kim et al.^20^ shows the clinical occurrence and impact of this complication.^21^ Retrospective assessment by the lead author of a group of patients with rotator cuff repair failure showed this manner of failure. In addition, intraoperative observations of bony collapse while inserting a lateral row anchor in “weak” bone led us to carry out this lab‐based biomechanical study.

A successful rotator cuff repair is multifactorial, and there are elements that we are still yet to properly understand, but looking through the available literature, it is evident that an adequate footprint coverage with prevention of gap formation is paramount.^1^, ^3^, ^13^, ^15^, ^26^ Integrity of the speed bridge rotator cuff repair is governed by the lateral row anchor, and regarding this,^5^, ^7^, ^8^, ^18^, ^19^, ^21^, ^23^ we believe that the type of suture used and the quality of bone have an independent and overlapping role to play, which has been shown in our research.

### Bone Density

Chung et al.,^14^ in their study to understand the independent effect of osteoporosis on rotator cuff healing, showed that patients with a low bone mineral density had a 7.25 times increased possibility of healing failure. The strength of rotator cuff repairs might be compromised by decreased bone quality, resulting in suture anchor loosening or pullout before adequate tendon‐to‐bone healing can occur.

In our observation, we noted that “weak” bone at the time of surgery is associated with a collapse of bone under the load of the sutures entering the lateral row anchor. This has also been shown within our lab‐based study—with low density bone models showing more cut‐through as compared with normal bone. It is our understanding that this collapse of bone leads to a loss of interference fixation of the suture between the bone and suture anchor, which correlates with our maximum force tolerance data.

Contrary to the popular “Deadman's angle” for anchor insertion, Nagamoto et al.^22^ showed that anchors inserted at 90° to the bone surface had better pullout strength when compared with anchors inserted at 45° when used in low density bone. Additionally, Wieser et al.,^24^ in their testing protocol, showed that suture slippage occurred at lower loads than anchor pullout when they tested knotless suture anchors. In a finite element analysis conducted by Sano et al.,^27^ they showed that stress concentration was most on the traction side (near side) of the anchor, and this reduced with changing the angle of pull and making it more in line with the insertion angle.

### Lateral Row Anchor

A multitude of anchor designs exists but functions on a similar basis—when inserted, there is a hold on the loaded suture through friction fit between the anchor eyelet and the suture, along with interference fixation of the suture between the bone and anchor. Some anchors have an additional locking design built in as well.^13^, ^23^, ^28^, ^29^

Wieser et al.^24^ showed that suture slippage in knotless anchors occurs at a much lower threshold load than anchor pullout/suture breakage/rotator cuff tendon cut‐through. This highlights the lateral row knotless anchor as being the weakest link in the chain. Additionally, they assessed the interaction of different anchor materials with different sutures and concluded that the geometric design of the clamping mechanism of the anchor might compensate for the relatively poor friction between the anchor and suture material.

This in turn shows that an effective lateral row anchor construct is one that maintains interference fixation of the suture along with friction fixation of the suture.

### Suture Material

Our study highlighted that high‐strength suture tape can withstand a higher maximum force and shows a lower displacement. Deranlot et al.^30^ and Wust et al.^11^ showed that braided polyblend sutures have more abrasive properties when tested on rotator cuff tendon and cartilage, respectively. Kowalsky et al.^31^ showed that the braided polyblend suture material was significantly more resistant but also more abrasive on tendon and bone compared with monofilament sutures. Borbas et al.,^32^ in their study, noted that high‐strength suture tapes provided significantly greater biomechanical strength and stiffness, potentially offering a more robust construct for rotator cuff repair. These higher suture resistance/abrasive properties might equate to an increased friction fixation between the suture, suture anchor, and bone in the lateral row and hence a higher maximum force.

Chuang et al.,^33^ in their study looking at ST, noted that there was a reduced abrasiveness which led to lower static friction at the tape‐anchor interface, which presented with a reduced clinical failure load. They also noted that doubling the number of FiberWire sutures passing through the lateral row anchor increased the clinical failure load, whereas doing the same with ST did not equate to a higher clinical failure load. This might be because of a lower frictional coefficient at the suturetape‐suturetape interface because of its coating.

### Limitations

This study is not without limitations. This laboratory‐based study employed synthetic Sawbones rather than cadaveric or clinical bone, which may not replicate the viscoelastic behavior of human tissue. Additionally, Sawbones immersion in saline and absence of a medial row construct restrict the model's clinical simulation.

## CONCLUSIONS

Suture slippage in knotless lateral row fixation is significantly influenced by the suture material and the underlying bone density. Constructs using high‐strength suture tape showed higher load‐to‐failure and lower displacement compared with conventional sutures.

## DISCLOSURES

The author (E.C.C.) declares the following financial interests/personal relationships which may be considered as potential competing interests: E.C.C. reports a relationship with The Lincoln Centre for bone and joint research that includes board membership and funding grants. The other authors (K.S., M.D., C.S., A.Y., B.C., M.M.S.) declare that they have no known competing financial interests or personal relationships that could have appeared to influence the work reported in this article.
